# Measuring hospital inpatient Procedure Access Inequality in the United States

**DOI:** 10.1093/haschl/qxae142

**Published:** 2024-11-06

**Authors:** Alon Bergman, Guy David, Ashwin Nathan, Jay Giri, Michael Ryan, Soumya Chikermane, Christin Thompson, Seth Clancy, Candace Gunnarsson

**Affiliations:** Department of Medical Ethics and Health Policy, University of Pennsylvania, Philadelphia, PA 19104, USA; Health Care Management Department, The Wharton School, University of Pennsylvania, Philadelphia, PA 19104, USA; Health Care Management Department, The Wharton School, University of Pennsylvania, Philadelphia, PA 19104, USA; Department of Medicine, University of Pennsylvania, Philadelphia, PA 194104, USA; Department of Medicine, University of Pennsylvania, Philadelphia, PA 194104, USA; MPR Consulting, Cincinnati, OH 45233, USA; Edwards Lifesciences, Irvine, CA 92614, USA; Edwards Lifesciences, Irvine, CA 92614, USA; Edwards Lifesciences, Irvine, CA 92614, USA; Gunnarsson Consulting LLC, Jupiter, FL 33477, USA

**Keywords:** access to care, health disparities, inpatient procedures, minimally invasive procedures, healthcare innovation, hospital market concentration

## Abstract

Geographic disparities in access to inpatient procedures are a significant issue within the US healthcare system. This study introduces the Procedure Access Inequality (PAI) index, a standardized metric to quantify these disparities while adjusting for disease prevalence. Using data from the Healthcare Cost and Utilization Project State Inpatient Databases, we analyzed inpatient procedure data from 18 states between 2016 and 2019. The PAI index reveals notable variability in access inequality across different procedures, with minimally invasive and newer procedures exhibiting higher inequality. Key findings indicate that procedures such as skin grafts and minimally invasive gastrectomy have the highest PAI scores, while cesarean sections and percutaneous coronary interventions have the lowest. The study highlights that higher inequality is associated with greater market concentration and in particular, fewer hospitals offering these procedures. These findings emphasize the need for targeted policy interventions to address procedural access disparities to promote more equitable healthcare delivery across the United States.

## Introduction

Clinicians, health policy experts, and regulators have been increasingly focused on the problem of disparities in access to procedures.^1-8^ This issue has significant implications for both individual health outcomes and systemic health inequality. Previous studies have found disparities in procedural access to be associated with gaps in insurance coverage, socioeconomic status, race and ethnicity, age, disability status, lack of regulatory oversight, geographical location, income, and language barriers.^5,9-24^

Access to inpatient procedures, in particular, is a critical component of high-quality healthcare systems, yet disparities persist.^25-27^ Quantifying and comparing these disparities across procedures are challenging due to differences in the underlying epidemiology and disease burden.^28^ We propose a novel metric, the Procedure Access Inequality (PAI) index, which provides a standardized measure of the inequality in geographical access to inpatient procedures while accounting for the inherent unequal geographical dispersion of disease.

Using an all-payer encounter-level database covering 18 US states from 2016 through 2019, we ranked the 40 highest-volume inpatient procedure categories according to their PAI index; this ranking highlights procedures with the greatest relative disparities in access after adjustment for disease prevalence. We then explored potential drivers of variable access inequality, including hospital availability, procedure characteristics, patient demographics, insurance coverage, and case volumes.

Finally, we conducted a detailed case study of aortic valve replacement procedures, comparing transcatheter aortic valve replacement (TAVR) and surgical aortic valve replacement (SAVR). This case study provides a valuable illustration of the dynamics of procedural access inequality for several reasons. First, it allows us to examine how access inequality evolves as a newer, minimally invasive procedure (TAVR) is introduced alongside an established surgical procedure (SAVR) for treating the same condition. Second, the rapid expansion of TAVR between 2016 and 2019 offers insight into how changes in procedure availability and hospital offerings can impact access inequality over a relatively short time period. Lastly, by analyzing these procedures both separately and in combination, we can assess whether the introduction of a new treatment option improves overall access to care for patients with aortic stenosis or potentially exacerbates existing disparities.

## Methods

### Data sources and sample

This study uses Healthcare Cost and Utilization Project (HCUP) State Inpatient Databases (SID) from the Agency for Healthcare Research and Quality (AHRQ). We collected 2016-2019 procedure volume data from the HCUP SID data for the 18 states that published patient zip code information: Arizona, Delaware, Florida, Indiana, Iowa, Kentucky, Maryland, Michigan, Minnesota, Missouri, New Jersey, North Carolina, Oregon, Rhode Island, South Dakota, Vermont, Washington, and Wisconsin. The states, which are drawn from all 4 US census regions, account for 111 million Americans.

The data contain the universe of hospital discharges in those states and years, regardless of patient coverage. For each discharge, we observe the patient's demographic characteristics (including age, sex, and race), their insurance coverage, their length of stay, the inpatient procedure associated with their hospital stay, and importantly, the zip code in which the patient resides when not in the hospital. This study further incorporated Census American Community Survey data providing 5-year population estimates at the zip code level, stratified by age group. Utilizing this granular demographic information allowed for age-adjusting the Procedure Access Inequality calculations across geographic areas.

### Inpatient procedures

Inpatient procedures in our data are described using ICD-10-PCS codes, which number over 80 000. To reduce this complexity, we used the Clinical Classifications Software Refined (CSSR), developed by AHRQ, to group inpatient hospital surgical procedures from individual ICD-PCS-10 codes into 320 clinically meaningful categories. Of those, 195 categories are associated with ICD-10-PCS codes that appear in the HCUP data. We further decompose these 195 CSSR categories by classifying them into either minimally invasive (MI) or non-minimally invasive (non-MI) based on the description of the underlying ICD-10-PCS code. However, many CSSR categories, like “Heart valve replacement and other valve procedures (endovascular),” were already defined based on this characteristic. After narrowing the set of ICD-10-PCS codes to those appearing in the HCUP data, aggregating these codes into CCSR categories, and then categorizing them as MI or non-MI, we ultimately arrive at 356 inpatient surgical procedure categories.

Two characteristics of surgical procedures we consider important to analyze—the year the procedure category was first introduced, and whether any procedure within the category involve the implantation of a device—are not available in the HCUP data. Since manually constructing these data for 356 inpatient procedure categories would require countless hours of work, we turned instead to generative AI models to establish these values. We derive information about the year the procedure category was first introduced, and whether any procedure within the category involves the implantation of a device, using the generative AI models GPT 4o and Claude Sonnet 3.5.^29^ Disagreements between the models were manually reviewed and settled.^30^

### PAI score

We introduce the PAI score as a standardized metric, ranging from 0 to 1. The PAI score quantifies the degree of geographical dispersion of inpatient procedures. This dispersion is measured by comparing the geographic distribution of patients receiving a particular procedure and the overall geographic distribution of the condition being treated. A procedure that is more unequally distributed geographically, relative to the distribution of the underlying condition it treats, would receive a higher PAI score. Procedure Access Inequality facilitates the direct comparison of inpatient procedures over time and across specialties, procedural approach, conditions treated, and all other measurable procedure characteristics.

We calculated the PAI score for each of our 356 inpatient procedure categories. The resulting PAI score ranges from 0, representing perfect equality (the distribution of patients receiving the procedure is identical to the distribution of all hospitalizations in the sample), to 1, representing perfect inequality (all the procedures were performed on patients residing in a single zip code in the sample).

The Online Appendix offers a technical discussion on the construction of the PAI measure. In summary, we start by calculating 2 statistical measures of dispersion for each procedure: the baseline dispersion and the realized overall dispersion. The baseline dispersion is influenced by area-level population characteristics (specifically age distribution by sex) and serves as a proxy for the prevalence of the condition treated by the procedure.^31^ In contrast, the realized overall dispersion is determined by the observed utilization of the procedure across areas. The PAI score is derived from the gap between these 2 dispersions, representing inequalities in access, capacity, and offerings.^32^

Another key measure in our analysis is the Herfindahl-Hirschman Index (HHI), a widely used indicator of market concentration. For each procedure category, the HHI is calculated as the sum of the squared market shares of all hospitals performing procedures in that category across all 18 states in our sample. This index reflects not only the number of hospitals offering the procedure category but also the degree of concentration of the procedure among a relatively small number of hospitals. For example, if a procedure category is performed equally across 40 hospitals, the HHI would be 250. If the number of hospitals doubles but the distribution of surgeries remains even, the HHI would drop to 125, indicating decreased market concentration. See the Online Appendix for a technical discussion.

### Statistical analyses

The main outcome for this analysis is the PAI score for each of the 356 inpatient procedure categories in our sample for each calendar year 2016-2019. We report on the top 40 most frequently occurring (based on 2019 volume) inpatient procedure categories, which account for almost 70% of all procedures in our sample and display their PAI ranking. We sort procedure categories from most unequally distributed to least.

Next, we examined the highest-volume (top quintile) procedure categories for 2019, focusing on the relationship between a procedure category's PAI score and the number of hospitals where it is performed. We plotted these PAI scores against the number of hospitals performing each procedure category and calculated the correlation between the two. Additionally, we assessed the correlation between the percent change in these measures from 2016 to 2019 using the same procedure categories.

Finally, we grouped all 356 inpatient procedure categories by their terciles based on the PAI score distribution. We then presented the tercile-specific means and SDs for potential factors that might explain variations in PAI score. These factors include the age of the procedure category, its cost and coverage, outcomes, and the demographics of patients undergoing these procedures.

## Results

Table 1 presents the 40 highest-volume inpatient procedure categories in our sample, ordered by decreasing PAI score. In other words, procedures at the top of the list are more unequally distributed across patients than those at the bottom. The most unequally distributed procedure categories in 2019 were skin graft, endarterectomy and embolectomy (carotid and other), minimally invasive gastrectomy, toe and mid foot amputation, minimally invasive pacemaker and defibrillator procedures, and endovascular heart valve procedures. The most equally distributed procedure categories were cesarean section, percutaneous coronary interventions (PCIs), minimally invasive cholecystectomy, femur fixation, colectomy, bone excision, and hip arthroplasty. Overall, cardiovascular procedures are more concentrated in the top half of the table, while orthopedic procedures are spread evenly between top and bottom.

**Table 1.** Procedure inequality across top procedure categories, 2019.

Rank	Procedure category	PAI score	Procedure count	Hospital count	Hospital HHI
1	Skin graft	0.374	46 777	820	167.5
2	Carotid endarterectomy and stenting	0.302	69 708	586	36.2
3	Gastrectomy (minimally invasive)	0.293	50 530	546	57.2
4	Embolectomy, endarterectomy, and related vessel procedures (non-endovascular; excluding carotid)	0.248	46 441	619	39.5
5	Toe and mid foot amputation	0.223	81 159	1065	24.1
6	GI system lysis of adhesions (minimally invasive)	0.223	39 867	986	30.2
7	Arthroplasty of other joint (excluding knee and hip)	0.223	67 811	945	30.6
8	Pacemaker and defibrillator procedures (minimally invasive)	0.220	40 339	593	44.3
9	Heart valve replacement and other valve procedures (endovascular)	0.218	35 694	276	105.9
10	Fixation of upper extremity bones	0.216	38 482	922	51.8
11	Heart valve replacement and other valve procedures (non-endovascular)	0.209	44 480	327	83.3
12	Lumbosacral nerve decompression	0.208	59 694	634	43.1
13	Bone fixation (excluding extremities)	0.204	57 458	641	68.2
14	Femur fixation (minimally invasive)	0.190	38 330	953	25.5
15	Muscle, tendon, bursa, and ligament excision	0.189	63 988	1099	32.1
16	Vessel repair and replacement	0.187	41 114	665	57.3
17	Hysterectomy	0.177	37 060	924	36.3
18	Appendectomy (minimally invasive)	0.166	40 739	1065	21.3
19	Salpingectomy	0.157	46 923	949	34.2
20	Vertebral discectomy	0.150	106 122	652	38.8
21	Spine fusion	0.138	277 232	659	39.6
22	Angioplasty and related vessel procedures (endovascular; excluding carotid) (minimally invasive)	0.136	154 191	746	33.6
23	Musculoskeletal procedures, NEC	0.133	87 664	1024	37.8
24	Saphenous vein harvest and other therapeutic vessel removal (minimally invasive)	0.133	65 378	487	51.0
25	Fallopian tube ligation and excision	0.132	66 309	898	33.8
26	Small bowel resection	0.132	45 709	1002	33.8
27	GI system lysis of adhesions	0.128	64 840	1048	29.8
28	Fixation of leg and foot bones	0.128	75 060	1014	37.8
29	Knee arthroplasty	0.120	257 701	1058	25.7
30	Abdominal wall repair (including hernia)	0.113	49 024	1050	30.3
31	Perineal muscle laceration repair (second degree obstetrical and other)	0.112	223 256	870	33.2
32	Coronary artery bypass grafts (CABGs)	0.111	132 288	331	50.2
33	Subcutaneous tissue and fascia excision	0.110	111 646	1216	26.2
34	Hip arthroplasty	0.109	252 234	1064	23.7
35	Bone excision	0.093	92 996	1072	34.8
36	Colectomy	0.087	79 132	1074	25.1
37	Femur fixation	0.083	78 620	1020	23.9
38	Cholecystectomy (minimally invasive)	0.069	90 273	1119	19.8
39	Percutaneous coronary interventions (PCIs) (minimally invasive)	0.028	207 005	618	28.3
40	Cesarean section	−0.039	362 243	864	31.6

This table shows the top procedure categories in the sample by number of procedures performed. Procedures are ranked by decreasing PAI score (most unequally distributed to least). HHI is calculated as the sum of the squared market shares of all hospitals performing procedures in that category. Source: Authors’ analysis of HCUP SID data for 2019.

Since PAI is a standardized score, we can directly compare access inequality across different procedure categories. Skin graft, at the top of Table 1, exhibits the highest PAI, making it the least equally distributed inpatient procedure category. The most equally distributed procedure on the list is cesarean section, with an estimated point estimate of −0.039, below the hypothetical minimal range of PAI.^33^ Within cardiovascular procedure categories, endovascular heart valve procedure's PAI score was 96.4% higher than that of coronary artery bypass graft (CABG), and 678.6% higher than that of PCIs. Procedure Access Inequality score further allows for the comparison of procedures that only differ by type of approach. Endovascular heart valve procedures, which primarily include TAVR procedures, had a PAI score that was 4.3% higher than that of non-endovascular heart valve procedures (primarily SAVR). Minimally invasive femur fixation had a PAI score 128.9% higher than that of invasive femur fixation.

Within the top 40 inpatient procedure categories listed in Table 1, the average procedure category volume was 95 633 (SD = 78 468) cases spanning, on average, 837 hospitals (SD = 245). More commonly performed procedure categories tend to have a lower PAI score. Finally, hospital concentration as measured by the HHI score was particularly high among 2 of the most unequally distributed procedure categories, skin graft and endovascular heart valve procedures.

**Figure 1.**
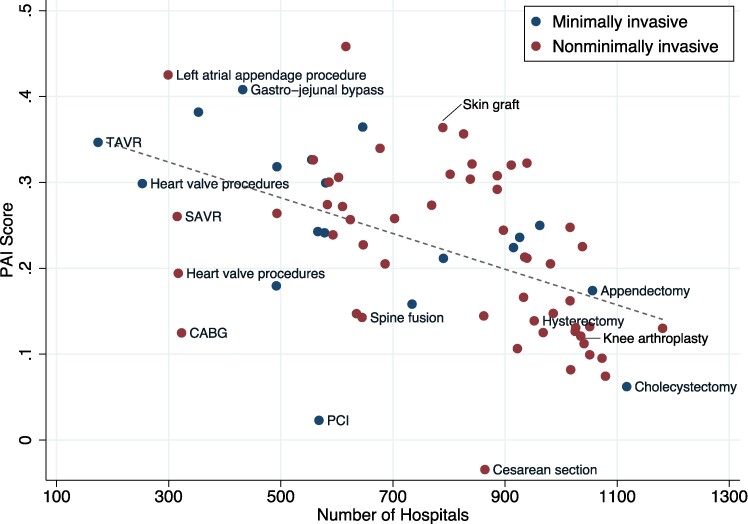
Procedure PAI score and number of performing hospitals, 2016. Source: Authors’ analysis of HCUP SID data for 2016. This figure presents a scatterplot of inpatient procedures by PAI score on the vertical axis, and number of procedure hospitals on the horizontal axis, using 2016 data. The dashed line represents the linear fit of the relationship.

In the secondary analysis, we expand the scope of our analysis to the top tercile (20%) of all inpatient procedure categories, by volume. Figure 1 provides a visual representation of the relationship between PAI score and the number of hospitals in 2016. Overall, we found a strong negative correlation between a procedure category's PAI score and the number of hospitals offering the procedure. A linear fit estimate suggests that an increase of 250 hospitals offering the procedure (approximately 1 SD) is correlated with a 0.042 (SE = 0.0047) decrease in PAI score—a reduction large enough to change a procedure category's relative ranking in Table 1 by several spots.

Cardiovascular procedures varied significantly by PAI score and number of hospitals performing the procedure. Transcatheter aortic valve replacement and SAVR, both used in the treatment of aortic stenosis, displayed divergent patterns. Transcatheter aortic valve replacement, now analyzed separately from other endovascular heart valve procedures, exhibited a high PAI score (0.347) and was offered in only 174 hospitals. Conversely, SAVR (now separated from non-endovascular heart valve procedures) exhibited a lower PAI score (0.260) and was offered in far more hospitals (315). Combined, aortic valve replacement therapy (SAVR and TAVR) exhibits a PAI score of 0.197 while being offered in 315 hospitals (see Online Appendix for figure).

Other cardiovascular procedures had a lower PAI score. For example, CABG procedures, though performed in a similar number of hospitals to SAVR, had a PAI score which was 47.9% lower than SAVR. Percutaneous coronary intervention exhibited the second lowest PAI score among the procedures in this set, after cesarean section, despite being offered in a smaller number of hospitals.

**Figure 2.**
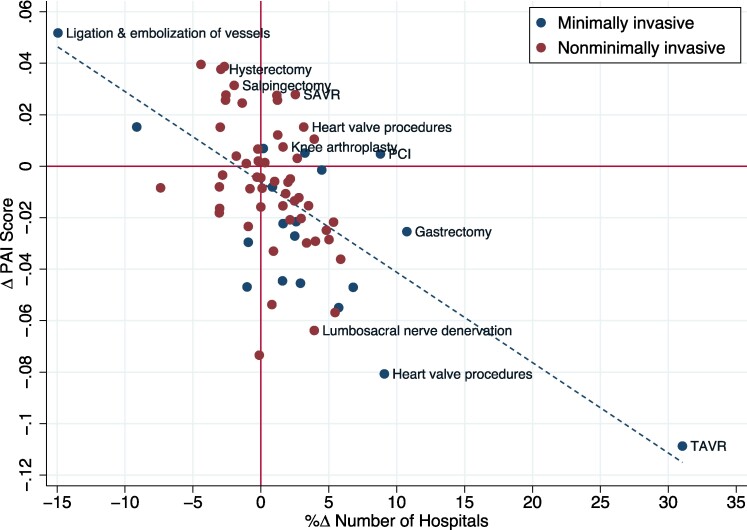
Percent change in procedure PAI score and in number of performing hospitals, 2019-2016. Source: Authors’ analysis of HCUP SID data for 2016 and 2019. This figure presents a scatterplot of inpatient procedures by percent change in PAI score on the vertical axis, and percent change in the number of procedure hospitals on the horizontal axis, from 2016 to 2019. The dashed line represents the linear fit of the relationship.

Figure 2 illustrates that the negative correlation between PAI score and the number of hospitals performing the procedure persists when studying changes in these measures over time.^34^ We measured the change in PAI score, on the vertical axis, compared to the percent change in the number of hospitals performing procedures in a given procedure category, on the horizontal axis, between 2016 and 2019. Similar to the cross-sectional analysis in Figure 1, the longitudinal analysis reveals a negative correlation between PAI score and hospital count. A univariate linear regression estimates that a 10% increase in the number of hospitals performing a given procedure is associated with a 0.035 reduction in the inequality in its provision.

The number of hospitals offering TAVR increased by 31% between 2016 and 2019. Over the same period, TAVR PAI score decreased by 31.4%, the largest decrease observed among all cardiac procedures. The number of hospitals offering ligation and embolization procedures decreased by 15% while incurring an increase of 24.5% in their PAI score. Other procedures experienced a significant increase in PAI score with only a small change in the number of hospitals. The surgical counterpart to TAVR, SAVR, experienced a 10.7% increase in PAI score during this period, while being performed in 2.5% more hospitals than in 2016. Combined, the PAI score of aortic valve replacement treatments decreased by 9.9%, accompanied with a 3.8% increase in the number of hospitals that offer treatment for aortic stenosis (see Online Appendix for figure).

Table 2 analyzes 19 characteristics across 356 procedure categories, further categorized by their 2019 PAI score terciles. Our key findings are that procedures with higher PAI scores—indicating greater access inequality—tend to involve younger patients, fewer White patients, and more Hispanic patients. These procedures also show lower Elixhauser comorbidity scores, with no significant differences in death rates or hospital stays. Additionally, patients undergoing procedures with higher PAI scores are less likely to have Medicare coverage, though procedure costs do not significantly differ across PAI scores. At the procedure category level, about 66% of high PAI procedures are minimally invasive, vs 29% in the lowest PAI tercile. These procedure categories are also more likely to be recent, with 42% of high PAI procedures being introduced in 1990s or later, and are likelier to require urgent/emergent admission. High PAI procedure categories are also less common, performed in fewer hospitals, and occur in more concentrated hospital markets.

**Table 2.** Summary statistics of potential factors affecting PAI in 2019, by procedure PAI score tercile.

	PAI range			*P*-value of difference
	(−0.04, 0.49)	(0.49, 0.84)	(0.84, 1.00)	
Procedure-patient level characteristics				
Average age	56.77	52.93	51.14	<0.001
	(9.30)	(10.36)	(12.07)	
% female	53.18	47.80	51.65	0.612
	(22.15)	(20.93)	(24.07)	
% White	71.95	69.92	69.26	0.044
	(8.59)	(9.81)	(11.62)	
% Hispanic	9.40	10.27	10.76	0.029
	(3.43)	(3.55)	(5.77)	
% Black	12.63	13.21	13.19	0.518
	(4.98)	(5.97)	(7.88)	
Elixhauser comorbidity score	3.13	3.07	2.80	0.030
	(1.15)	(1.19)	(1.16)	
Death rate	2.51	2.93	2.50	0.984
	(3.35)	(4.30)	(5.13)	
Length of stay (days)	8.37	10.08	9.65	0.193
	(5.08)	(7.42)	(9.42)	
Cost ($1000s)	37.16	47.56	45.11	0.134
	(20.73)	(44.45)	(53.63)	
% Medicare	42.93	38.80	36.90	0.005
	(16.32)	(14.83)	(16.73)	
% private	35.31	36.14	36.45	0.456
	(11.09)	(9.01)	(12.40)	
Distance traveled (miles)	32.38	41.07	42.08	<0.001
	(10.62)	(14.20)	(24.66)	
Procedure level characteristics				
% elective	50.68	53.41	49.07	0.637
	(26.45)	(25.70)	(25.99)	
% minimally invasive	30.25	43.70	67.23	<0.001
	(46.13)	(49.81)	(47.14)	
% device implant	29.06	22.69	26.32	0.643
	(45.60)	(42.06)	(44.23)	
% newer technology	19.66	26.05	41.59	<0.001
	(39.91)	(44.08)	(49.51)	
Procedures performed	42 876.37	3716.04	434.45	<0.001
	(59 061.61)	(2276.46)	(391.60)	
Number of hospitals	734.55	419.64	133.39	<0.001
	(225.61)	(150.23)	(104.31)	
HHI	52.99	104.29	589.32	<0.001
	(35.38)	(67.21)	(1183.81)	
Procedures	119	119	118	

This table presents means and summary statistics (in parentheses) for 19 procedure characteristics, when binning all procedure categories by the terciles of the PAI score distribution. *P*-value of difference is estimated based on the difference between the top and bottom PAI terciles. HHI is calculated as the sum of the squared market shares of all hospitals performing procedures in that category. Source: Authors’ analysis of HCUP SID data for 2019. GPT 4o and Claude Sonnet 3.5 were utilized for device implant and new technology information.

## Discussion

Access to inpatient procedures is not equally distributed in the United States. Our results identify large variability in the distribution and concentration of procedures, even across procedure categories that are of similar volume. Inequality in procedural access was not limited to procedures performed by a specific specialty, nor to those treating a specific medical condition. Instead, they ranged across specialties, from plastic surgery and gastrointestinal surgery to cardiac surgery, and interventional cardiology, among others. Overall, we found that procedures that are minimally invasive or are newer procedures that were introduced in the 1990s or later were likely to be more unequally distributed.

Access to medical care is complex and multifaceted. This includes socioeconomic, financial, and logistic issues for patients, their communities, and their healthcare systems.^35-38^ We found evidence of multi-level contributions to procedural access inequality. At the patient level, increases in the necessary distance required to travel for invasive procedures were associated with increased procedural inequality. This may be particularly relevant for socioeconomically disadvantaged patients who face transportation barriers and subsequent harm.^39-43^ At the physician level, minimally invasive or newer procedures introduced in the 1990s or later were associated with increased procedural inequality. Minimally invasive and newer procedures require specialized training.^44-46^ These techniques may be concentrated among fewer surgeons with these unique skills, and due to their limited numbers, may limit procedural access to geographic areas where these physicians practice.^10,47,48^ Thus, skilled workforce limitations could be significantly contributing to higher PAI, particularly among less mature procedures. Finally, at the health-system level, we found a strong association between fewer numbers of hospitals offering procedures and increasing procedural access inequity. In the case of TAVR, with increases in the number of hospitals offering the procedure over time, inequality decreased. Limitations on the number of sites offering procedures, as well as their geographic locations, can significantly affect patient access to invasive procedures.

One potential explanation for the variability in access inequality across inpatient procedures may be regional differences in the prevalence of the conditions they treat.^49,50^ If the prevalence of certain conditions varies across areas, apparent access inequality may instead reflect prevalence differences. Our prevalence-adjusted PAI measure directly accounts for this heterogeneity. Moreover, if variability were due to inequality in the underlying condition, we would expect little temporal variation in access inequality. We find evidence to the contrary; there is variation in PAI score even over horizons too short for the prevalence of a condition to change significantly. This suggests that changes in access over time are indeed significant, as illustrated by the case of TAVR. If this variation reflected only the prevalence of conditions, the number of hospitals performing the procedures would be less consequential. However, our analysis reveals that PAI score is strongly negatively correlated with the number of hospitals that perform the procedure, further suggesting that the variation is related to access rather than prevalence.

Focusing on TAVR as a case study, we observed that in 2016 TAVR was offered in fewer hospitals compared to other cardiovascular procedures and had the highest access inequality score. From 2016 to 2019, the number of hospitals performing TAVR increased by over 30%, the most significant expansion among the procedures studied, accompanied by a 31.4% decrease in access inequality—the largest decrease observed. In contrast, PCI was the second most equitably distributed procedure. The disparity in access between TAVR and PCI may stem from the more complex treatment-referral pathway, differences in training and skill requirements, the urgency of treating conditions, and the broader health-system adoption, with fewer facilities offering TAVR compared to PCI.^51^

The TAVR case study offers an opportunity to examine access inequality by condition rather than by procedure. As TAVR and SAVR are the only treatments for aortic stenosis, examining them together allows us to explore whether patients who need this life-saving intervention are able to access any treatment at all. Our findings reveal that while the combined PAI score for both procedures is lower than for either procedure individually, the reduction is not significant enough to indicate that both treatments jointly resolve access inequality. Furthermore, the large expansion in TAVR offering between 2016 and 2019 has reduced aortic stenosis access inequality overall. Unless aortic stenosis changed in prevalence or distribution during this period, the explanation for this decrease in PAI should likely relate to the regulatory and operational aspects of expanding the number of hospitals offering these procedures, and would suggest overall welfare gains for the population suffering from aortic stenosis.

The PAI index may serve as a useful measure in efforts to reduce access inequality. Given multi-level contributions to inequality, it is unlikely that there exists any 1 solution to improve patient access to care. Instead, multiple interventions are likely required to incrementally improve and promote the equitable delivery of health care in the United States.^52-54^ Interventions at the patient-level (eg, transportation vouchers, reduced copays, and patient navigators), community-level (eg, community outreach, infrastructure development, and reductions in poverty), health system-level (eg, established referral networks and system integration, physician recruitment, and changes in coverage determinations), and national societal-level (eg, medical education, training, and proctorship programs for physicians in practice designed to improve facility with novel techniques/procedures) will each have their role, and can be critically appraised using the PAI as a measure of interventional success.

Finally, we emphasize that achieving the lowest possible PAI score is not necessarily a desirable policy goal for all procedures. The optimal level of procedure distribution involves balancing equity, efficiency, and quality of care. While offering all surgical services in every hospital would reduce PAI scores, it would do so at a prohibitively high cost and potentially compromise quality for complex procedures. Conversely, the current limited distribution of many procedures often represents an extreme inequality in access.

The ideal distribution may vary depending on the procedure's nature and maturity. Newer or highly specialized interventions may initially benefit from concentration in select centers, gradually dispersing as they become more established, as illustrated by the TAVR case in our study. For common or less complex procedures, wider initial distribution may be more appropriate.

Therefore, the interpretation and targeted level of PAI should be procedure-specific and dynamic over time. The optimal level hinges on balancing equity and efficiency, considering factors such as procedure complexity, required expertise, and potential for quality improvement through volume-outcome relationships. This balance should be guided by policymakers, informed by clinical expertise and the broader social contract to provide equitable healthcare access.

## Conclusions

Novel standardized measurement reveals high geographical variability in access to inpatient procedures performed across 18 US states. Minimally invasive and novel procedures were more unequally distributed across zip codes. Access inequality is not static. Our results show that inequality can vary over time and is highly correlated with the number of hospitals which offer the procedure. The case of TAVR demonstrates that a significant, policy induced, increase in the number of hospitals offering a procedure can significantly affect its geographic access inequality. Policymakers should consider the potential dynamic when considering regulation affecting hospital procedure offerings.

## Supplementary Material

qxae142_Supplementary_Data

## Data Availability

The primary data source for this study, the Healthcare Cost and Utilization Project (HCUP) State Inpatient Databases (SID), is available through the Agency for Healthcare Research and Quality (AHRQ). Researchers can request access to these data through the HCUP Central Distributor. Due to data use agreements and patient privacy protections, these data cannot be shared directly by the authors. Information about requesting access is available at https://www.hcup-us.ahrq.gov/databases.jsp. Supporting demographic data from the Census American Community Survey 5-year population estimates are publicly available through the U.S. Census Bureau website (https://www.census.gov/programs-surveys/acs). Code for reproducing the PAI calculations and analyses, as well as the procedure categorization data generated using GPT-4o and Claude Sonnet 3.5, are available from the corresponding author upon request.
