# Supplementary material for: Measuring hospital inpatient Procedure Access Inequality in the United States
Source: Health Aff Sch. 2024 Nov 6;2(11):qxae142. doi: 10.1093/haschl/qxae142 (PMC11574731; doi:10.1093/haschl/qxae142)
Supplement: qxae142_Supplementary_Data [file qxae142_supplementary_data.zip › PAI Supplementary material.pdf]

**Measuring Hospital Inpatient Procedure Access Inequality in the United States**

Online Appendix

A1. Constructing a PAI Score

The PAI score builds upon the Gini coefficient, an economic measure of statistical dispersion typically used to measure the income or wealth distribution of a nation's residents. The Gini coefficient measures inequality in the distribution of wealth (or income) using the cumulative distribution curve of wealth in the population (known as the Lorenz Curve), as seen in Figure A1.

**Figure A1: Gini Coefficient Calculation Illustration**

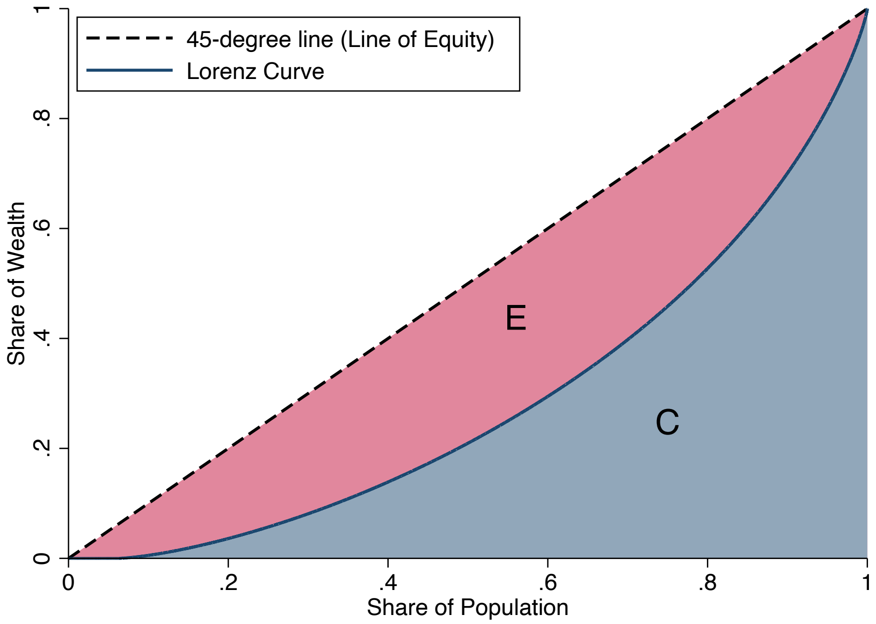


In Figure A1, the 45-degree line represents the Line of Equity – the line that the Lorenz curve would track were wealth perfectly equally distributed in the population. Given a Lorenz curve plot, the Gini coefficient is defined as twice the area between the Line of Equity and the Lorenz Curve (2 times E in the figure, or E divided by E+C). The resulting coefficient ranges between zero and one, with higher values reflecting more wealth inequality. At the extremes, wealth is either spread uniformly across the population (E= 0 ⇒ Gini = 0) or is concentrated completely in just one person (E = 0.5 ⇒ Gini = 1).

The PAI score can also be measured using calculations of area. Unlike Gini, PAI measures the share of inpatient procedures against the total share of hospitalizations in our sample. Instead of sorting individuals from lowest income to highest income, we sorted zip codes by the share of actual realized procedures (of a given kind) against the total number of hospitalizations in the zip code. In Figure A2, region C is the area below the realized distribution curve.[i] If the need for a procedure were equally distributed across all zip

codes in our sample, we would then measure inequality in a similar fashion to the Gini definition, by defining PAI as A+B. Morbidity, however, is not distributed equally across zip codes, and that inequality likely translates to unequal provision of the inpatient procedure aimed to treat it. We address this concern by approximating the distribution of the underlying condition requiring the procedure. We do so by estimating the average utilization of each procedure by age group and sex, and then predicting the expected baseline utilization in each zip code, based on its observed sex-specific age group distribution as recorded in the 2019 US Census American Community Survey 5-year estimates. We then plot the resulting baseline cumulative distribution, as illustrated in Figure A2. The PAI score can then be measured as B divided by B+C (or B divided by 2+A), which accounts for the fact that the Line of Equity is the baseline cumulative distribution curve, and not the 45-degree line.

**Figure A2: PAI Score Calculation Illustration**

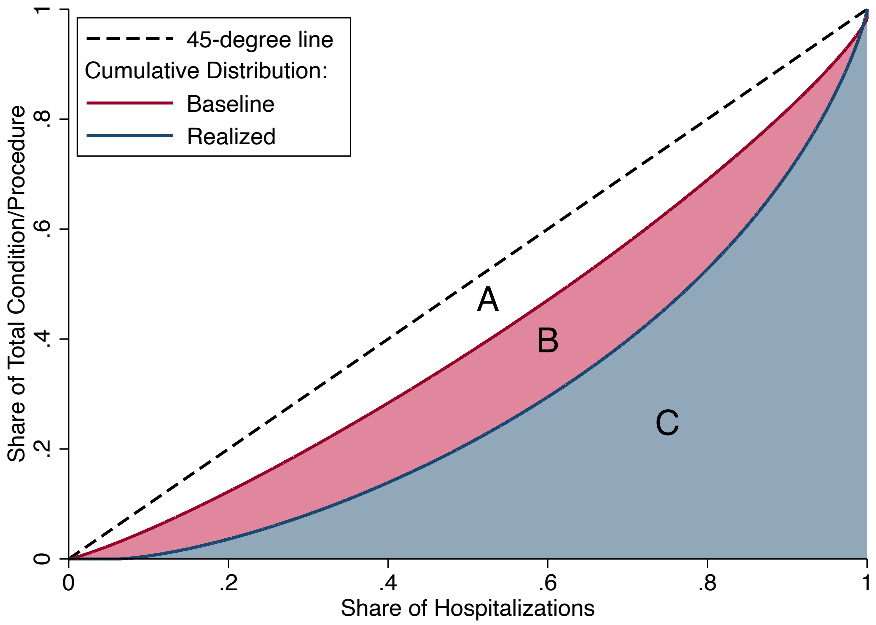


In practice, we measured PAI directly using a weighted ratio of $G_{Realized}$ and $G_{baseline}$, the two Gini coefficients described. We begin by calculating each Gini coefficient separately, using the grouped Gini coefficient formula described in Van Ourti and Clarke.[ii] let $n_u$ be the number of patients from zip code u ($u = 1, \dots K$) undergoing any inpatient hospitalization, and $p_u$ be the number of patients in the zip codes undergoing the procedure. Define $R_u = (n)^{-1}(\frac{1}{2n_u} + \sum_{j=1}^{u-1} n_j)$ as the fractional rank of zip code u, when sorting zip codes by $p_u/n_u$. The variance of the fractional rank is defined by $\sigma_{R_u}^2 = (n)^{-1} \sum_{u=1}^{K} n_u (R_u - 1/2)^2$. Then, the adjusted Gini score of the realized procedure distribution p, $G_{Realized}$, is given by the coefficient $\beta$ in estimating the following WLS regression:

$$2\sigma_{R_u}^2 \frac{p_u}{\bar{p}} \sqrt{n_u} = \alpha\sqrt{n_u} + \beta R_u\sqrt{n_u} + \epsilon_u\sqrt{n_u}$$

Calculating $G_{baseline}$ in an identical fashion, it can be shown that, for a given procedure:

$$PAI = \frac{G_{Realized} - G_{Baseline}}{1 - G_{Baseline}} = \frac{B}{B + C}$$

Which we calculate separately for each procedure and year in our sample.

A2. Constructing an HHI score

The Herfindahl-Hirschman Index (HHI) is used in this study as a measure of market concentration for hospitals performing specific inpatient procedures. The HHI is calculated as the sum of the squared market shares of all hospitals performing a given procedure. For each procedure, the market share of a hospital is defined as the percentage of the total procedure volume performed by that hospital relative to the total volume across all hospitals in the sample.

Mathematically, the HHI is expressed as:

$$HHI = \sum_{i=1}^{N} (s_i)^2$$

Where:

- N is the total number of hospitals performing the procedure in all 18 states in our sample.
- $s_i$ is the market share of hospital $i$, expressed as percentage of total procedure volume.

The HHI ranges from 0 to 10,000, where an HHI of 0 indicates perfect competition (many hospitals performing the procedure with equal shares), and an HHI of 10,000 indicates a monopoly (one hospital performing 100% of the procedures). For example, if a procedure is performed equally across 40 hospitals, each hospital would have a market share of 2.5%, and the HHI would be calculated as:

$$HHI = 40 \times (2.5)^2 = 250$$

In our analysis, higher HHI values correspond to a greater concentration of procedures being performed by a smaller number of hospitals, reflecting less competition and potentially more restricted access to the procedure. Conversely, lower HHI values indicate that the procedure is more evenly distributed across hospitals.

A3. Visual Representation of Procedural Access Inequality with Aortic Valve Replacement (AVR) Markers

In this appendix, we present Figures 1 and 2 again, this time with an additional marker representing the total Aortic Valve Replacement (AVR) procedures, which combines both Transcatheter Aortic Valve Replacement (TAVR) and Surgical Aortic Valve Replacement (SAVR) procedures.

**Figure A3: Procedure PAI Score and Number of Performing Hospitals of Aortic Valve Replacement Procedures, 2016**

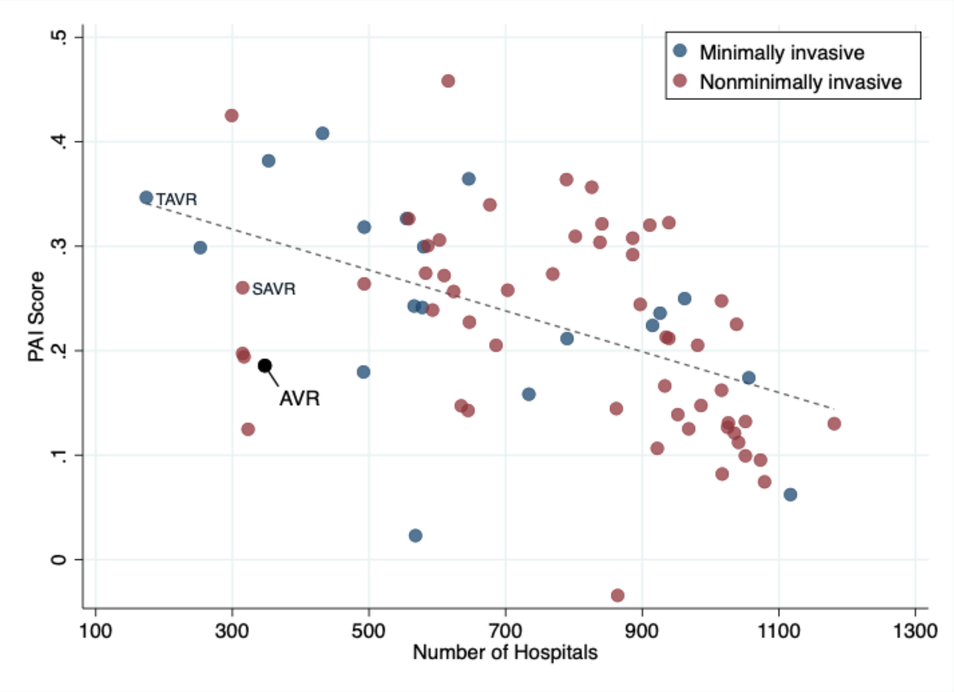


SOURCE Authors' analysis of HCUP SID data for 2016. NOTES This figure presents a scatterplot of inpatient procedures by PAI score on the vertical axis, and number of procedure hospitals on the horizontal axis, using 2016 data. The dashed line represents the linear fit of the relationship.

**Figure A4: Percent Change in PAI Score and Percent Change in Number of Performing Hospitals, Aortic Valve Replacement Procedures (2016-2019)**

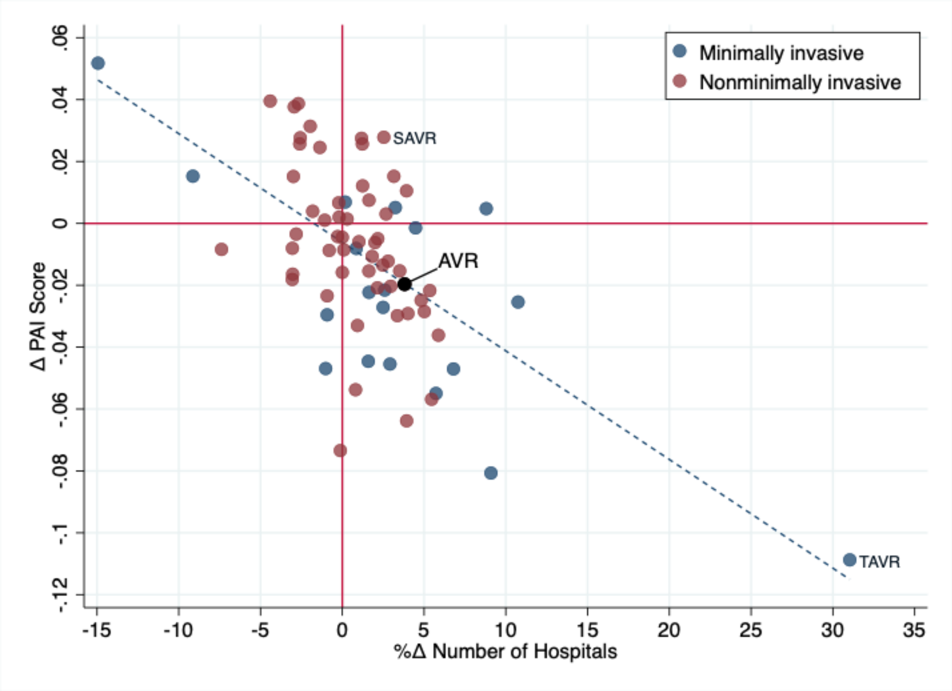


SOURCE Authors' analysis of HCUP SID data for 2016 and 2019. NOTES This figure presents a scatterplot of inpatient procedures by percent change in PAI score on the vertical axis, and percent change in the number of procedure hospitals on the horizontal axis, from 2016 to 2019. The dashed line represents the linear fit of the relationship.

---

[i] In practice, we perform this process separately for each procedure-year

[ii] Van Ourti T, Clarke P. A simple correction to remove the bias of the Gini coefficient due to grouping. Review of Economics and Statistics. 2011 Aug 1;93(3):982-94. doi:10.1162/REST_a_00103
